# Two Different Epidemiological Scenarios of Border Disease in the Populations of Pyrenean chamois (*Rupicapra p. pyrenaica*) after the First Disease Outbreaks

**DOI:** 10.1371/journal.pone.0051031

**Published:** 2012-12-10

**Authors:** Laura Fernández-Sirera, Oscar Cabezón, Alberto Allepuz, Rosa Rosell, Cristina Riquelme, Emmanuel Serrano, Santiago Lavín, Ignasi Marco

**Affiliations:** 1 Servei d’Ecopatologia de Fauna Salvatge (SEFaS), Facultat de Veterinària, Universitat Autònoma de Barcelona, Bellaterra, Barcelona, Spain; 2 Centre de Recerca en Sanitat Animal (CReSA), Universitat Autònoma de Barcelona, Bellaterra, Barcelona, Spain; 3 Departament de Sanitat i Anatomia Animals, Universitat Autònoma de Barcelona, Barcelona, Spain; 4 Departament d’Agricultura, Alimentació i Acció Rural, Generalitat de Catalunya, Barcelona, Spain; 5 Estadística i Investigació Operativa, Departament de Matemàtica, Universitat de Lleida, Lleida, Spain; Nanyang Technological University, Singapore

## Abstract

Since 2001 several outbreaks of a new disease associated with *Border disease virus* (BDV) infection have caused important declines in Pyrenean chamois (*Rupicapra pyrenaica*) populations in the Pyrenees. The goal of this study was to analyze the post-outbreak BDV epidemiology in the first two areas affected by disease with the aim to establish if the infection has become endemic. We also investigated if BDV infected wild and domestic ruminants sharing habitat with chamois. Unexpectedly, we found different epidemiological scenarios in each population. Since the disease outbreaks, some chamois populations recuperated quickly, while others did not recover as expected. In chamois from the first areas, prevalence was high (73.47%) and constant throughout the whole study period and did not differ between chamois born before and after the BDV outbreak; in all, BDV was detected by RT-PCR in six chamois. In the other areas, prevalence was lower (52.79%) and decreased during the study period; as well, prevalence was significantly lower in chamois born after the disease outbreak. No BDV were detected in this population. A comparative virus neutralisation test performed with four BDV strains and one *Bovine viral diarrhoea virus* (BVDV) strain showed that all the chamois had BDV-specific antibodies. Pestivirus antibodies were detected in all the rest of analyzed species, with low prevalence values in wild ruminants and moderate values in domestic ruminants. No viruses were detected in these species. These results confirm the hypothesis that outbreaks of BDV infection only affect the Pyrenean chamois, although other wild ruminants can occasionally be infected. In conclusion, two different scenarios have appeared since the first border disease outbreaks in Pyrenean chamois: on the one hand frequent BDV circulation with possible negative impact on population dynamics in some areas and on the other, lack of virus circulation and quick recovery of the chamois population.

## Introduction

The Pyrenean chamois *(Rupicapra pyrenaica pyrenaica)* is a mountain ungulate endemic to the Pyrenees (Northern Spain, Andorra and Southern France) that belongs to the order *Artiodactyla*. In 2001 and 2002 an outbreak of a previously unreported disease associated with BDV infection was described in Pyrenean chamois from the Central Pyrenees, specifically in the Alt Pallars-Aran National Hunting Reserve (NHR). Phylogenetic analysis of the 5′UTR region typed the chamois pestivirus as BDV-4 genotype [1]–[2]. After the outbreak, the population was found to have decreased by about 42%, most probably due to the disease. This was the first time that a BDV had been associated with an outbreak of a high-mortality disease in a wild species and the clinicopathological aspects were described [3]. Subsequently, several disease outbreaks associated with the same virus occurred in other Pyrenean chamois populations [4].

Along with *Bovine viral diarrhoea virus* 1 and 2 (BVDV-1 and -2) and *Classical swine fever virus* (CSFV), BDV belongs to the genus *Pestivirus* (family *Flaviviridae*). Ruminant pestiviruses (BDV and BVDV) are not strictly host-specific and transmission between different Artiodactyla species has been widely described [5]–[6]. BDV is distributed worldwide and causes disease mainly in sheep, but also in goats [7]. Postnatal infection in sheep tends to be mild and is characterized by mild pyrexia and transient lymphopaenia, followed by seroconversion [7]. However, severe outbreaks of disease with high mortality have been reported occasionally in cases of acute BDV infections in sheep [8]; as well, a mucosal disease syndrome has been described in persistently infected (PI) sheep [9].

Like all pestiviruses, BDV has the ability to pass through the placenta and infect the foetus with varying consequences. In sheep, if infection occurs before day 60 of the gestation period (i.e. before foetal immunocompetence) and if the foetus survives, the newborn will be a PI animal characterized by specific immunotolerance against BDV, an absence of antibodies and the continuous shedding of the virus throughout its life. PI animals can appear normal but usually grow poorly and have lower life expectancy [6]. PI individuals play a crucial role in maintaining pestiviruses in a flock.

After a decade of disease outbreaks in Pyrenean chamois populations, several questions remained unanswered. Marco *et al*. showed in 2008 [10] that this infection had become endemic in the Alt Pallars-Aran NHR, two years after the first disease outbreak. The goal of the present study was to analyze in the long term the post-outbreak BDV epidemiology in the first two areas affected by disease with the aim to establish if the infection has become endemic. In addition, we investigated if BDV infected wild and domestic ruminants sharing habitat with chamois. With this work both aims were successfully achieved.

## Materials and Methods

### Study area

**Figure 1:**
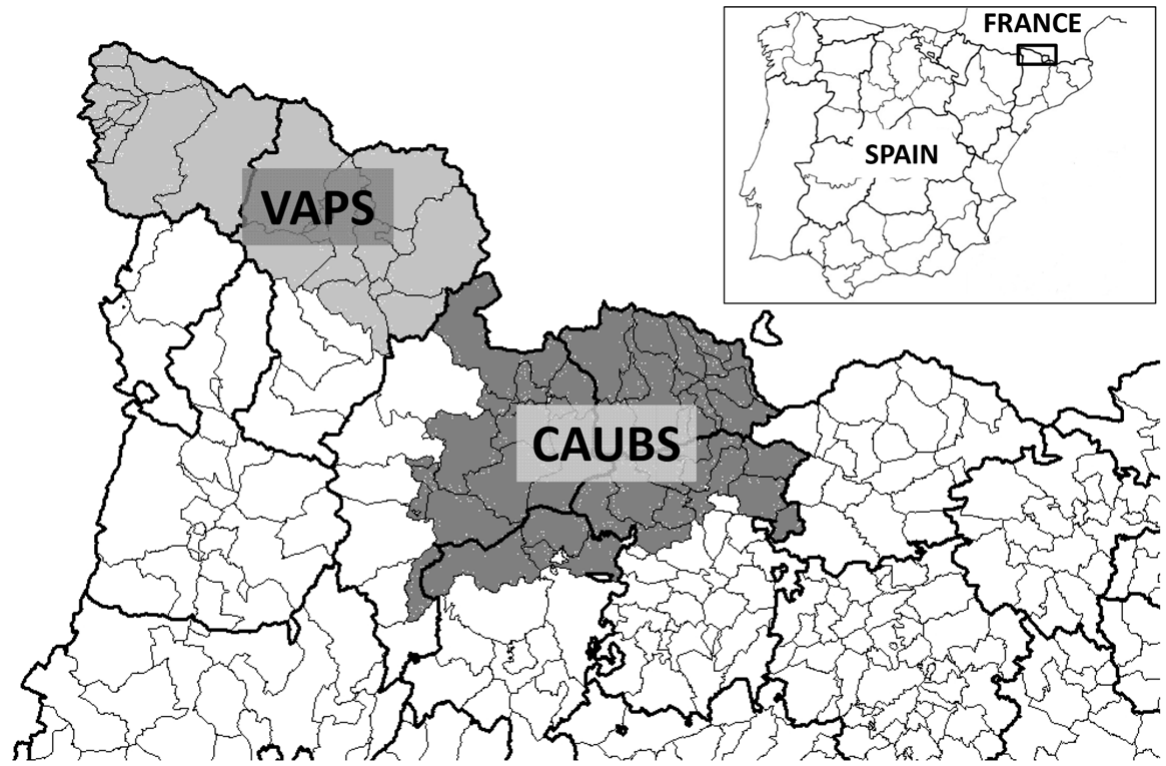
Study area. Location of the study areas both situated in the Spanish Pyrenees: Val d’Aran and Pallars Sobirà = VAPS; Cerdanya, Alt Urgell, Berguedà and Solsonès = CAUBS (Spanish Pyrenees). Dark lines represent regional boundaries and thin lines municipal boundaries.

The presence and epidemiology of BDV in ruminant populations was studied in two areas of the Pyrenees (Figure 1), both in the central Catalan Pyrenees (NE Spain, 1°15′N, 42°37′E) on the border with France. The first study area consists of the regions of Val d’Aran and Pallars Sobirà (VAPS), which includes most of the Alt Pallars-Aran NHR and adjacent private hunting areas (HPA). The disease was described for the first time in this area and between 2001 and 2002 caused an estimated decrease in the local chamois population of 42% [3]. After this outbreak, the population continued to fall, dropping from 3,526 chamois in 2003 to 2,441 chamois in 2011. The second study area is situated in the Eastern Pyrenees in the regions of Cerdanya, Alt Urgell, Berguedà and Solsonès (CAUBS), and includes the Cadí and Cerdanya-Alt Urgell NHR and adjacent HPA. During 2005, a disease outbreak led to the collapse of the chamois population in the Cerdanya-Alt Urgell NHR, causing an estimated cumulative rate of decline of 85.6%. In June 2005, the disease spread to the Cadí NHR and private hunting areas, with a subsequent estimated cumulative rate of fall of 63% [4]. Nevertheless, after these outbreaks, chamois populations have recovered successfully in this latter area, rising from 133 chamois in 2006 to 384 chamois in 2011 in the Cerdanya-Alt Urgell NHR and from 1,224 chamois in 2007 to 2,066 chamois in 2011 in the Cadi NHR (Direcció General de Medi Natural i Biodiversitat, Generalitat de Catalunya).

Pyrenean chamois share habitat with other wild ruminant species. Roe (*Capreolus capreolus*), red (*Cervus elaphus*) and fallow (*Dama dama*) deer, along with European mouflon (*Ovis aries*) inhabit VAPS, while roe and red deer live in CAUBS. As well, both study areas are characterized by communal alpine pastures that are shared by livestock (sheep, goats and cattle). Communal alpine pasturing is a centuries-old farming practice that involves the pasturing of domestic ruminants from different farms on grassland at high altitude in the Pyrenees, generally from May or June through to September.

### Ethics Statements

This study is part of a project (reference: CGL2006-11518/BOS and CGL2009-09071/BOS) from the Spanish Government which aims to study the epidemiology of pestiviruses in wild and domestic animals from Catalonia, a region from Spain. The sampling system used in the present study was approved by the Government in the framework of the project cited above.

In this work, both wild and domestic ruminants were sampled. Regarding to wild ruminants, samples were obtained from legally hunted dead animals. No protected species were sampled. The animals were collected during routine hunting in different National Hunting Reserves (NHR) (which are property of the Government) and they were not hunted expressly for this study. Therefore there was not necessary any ethics committee approval. The samples were collected by rangers employed by the Catalan Government. The rangers accompany the hunters and make a strict control of which animals are hunted. The sampling was performed within the framework of an official agreement between the Departament d’Agricultura, Ramaderia, Pesca, Alimentació i Medi Natural of the Generalitat de Catalunya and Universitat Autònoma de Barcelona. This agreement has as a main goal the surveillance of diseases from wildlife from the region of Catalonia, in Spain. The permission to use the samples is also included in this agreement. After the sampling, samples were delivered to our laboratory by government employers.

Regarding to domestic animals, blood samples were obtained during the annual compulsory sanitary campaigns by veterinarians from the Catalan Government. These campaigns are obligatory and the owner consent is not necessary. These campaigns are performed in order to survey the presence of diseases subjected to official eradication programs. Sera aliquots of the samples were delivered to our laboratory by veterinarians of the Government in order to analyze the presence of pestiviruses in the same framework of the agreement and the project cited above.

### Animals and Samples

Samples from apparently (clinically) healthy wild ruminants were obtained during the hunting season (HS) (September to February) over a five-year period between 2007 and 2011. In VAPS we studied samples from 114 Pyrenean chamois, 52 roe, 43 red and 25 fallow deer, and six mouflons. In CAUBS we studied samples from 115 Pyrenean chamois and 29 roe and 27 red deer (Table 1). Samples consisted of sera and/or spleen tissue. Blood samples, obtained by intracardiac venipuncture from animals that had been hunted, were placed into sterile serum separator tubes and centrifuged at 1200 g for 15 minutes. Spleen tissue was also collected. Sera and spleen samples were stored at −20°C until analysis.

**Table 1:** Distribution of wild ruminant samples by year and species.

STUDY AREA	SPECIES	2007	2008	2009	2010	2011	TOTAL OF TESTED ANIMALS
		n = 9	n = 13	n = 23	n = 21	n = 48	
VAPS	Pyrenean chamois chamois	8 spleen	8 spleen	16 spleen	17 spleen	24 spleen	114
		8 sera	13 sera	17 sera	20 sera	41 sera	
		n = 5	n = 9	n = 23	n = 1	n = 14	
VAPS	Roe deer	1 spleen	4 spleen	15 spleen		11 spleen	52
		5 sera	9 sera	20 sera	1 serum	12 sera	
		n = 3	n = 9	n = 21	n = 10	n = 0	
VAPS	Red deer	3 spleen	4 spleen	16 spleen	7 spleen		43
		3 sera	9 sera	21 sera	10 sera		
		n = 1	n = 19	n = 4	n = 1	n = 0	
VAPS	Fallow deer	1 spleen	16 spleen	4 spleen	1 spleen		25
		1 serum	15 sera	4 sera	1 serum		
		n = 0	n = 1	n = 2	n = 3	n = 0	
VAPS	Mouflon		1 spleen	2 spleen	3 spleen		6
			1 serum	2 sera	3 sera		
		n = 5	n = 26	n = 30	n = 39	n = 15	
CAUBS	Pyrenean chamois	5 spleen	23 spleen	29 spleen	37 spleen	15 spleen	115
		5 sera	23 sera	26 sera	38 sera	15 sera	
		n = 4	n = 4	n = 7	n = 6	n = 8	
CAUBS	Roe deer	4 spleen	4 spleen	7 spleen	6 spleen	7 spleen	29
		3 sera	3 sera	4 sera	5 sera	7 sera	
		n = 2	n = 6	n = 7	n = 12	n = 0	
CAUBS	Red deer	2 spleen	5 spleen	7 spleen	12 spleen		27
		2 sera	4 sera	7 sera	12 sera		

n = number of tested animals.

VAPS = Val d’Aran and Pallars Sobirà regions.

CAUBS = Cerdanya, Alt Urgell and Berguedà regions.

Sera from domestic ruminants were obtained during annual sanitary campaigns conducted when livestock descend from summer pastures. Blood samples were obtained by jugular venipuncture. In VAPS we obtained samples over a period of four years (2007–2010) from 360 sheep, 258 goats and 361 cattle, while in CAUBS we obtained samples from just two years (2007 and 2008) from 184 sheep, 96 goats and 175 cattle.

### Serological Tests

Sera were tested for the presence of antibodies against pestivirus with a commercial blocking ELISA assay (BVD/MD/BD P80, Antibody Screening, Pourquier, Montpellier, France). This test detects antibodies against the NS3 protein, common to all pestiviruses, and has a minimum specificity of 99.2% and an observed sensitivity of 100% for testing BDV positive sheep sera [11].

In order to confirm the results of the ELISA test and to determine the specificity of the antibodies, part (depending on the sample availability) of the selected positive ELISA sera were subsequently tested with a comparative virus neutralization test (VNT) using BVDV-1 strain NADL [12]; (Gen Bank accession number M31182), BDV-1 strain 137/4 [13]; (Gen Bank accession number U65052) and BDV-4 strain Esp97 (Gen bank accession number FR714860). Sera from VAPS were also tested with BDV-4 strain Aran-1 [10]; (Gen Bank accession number AM765800) isolated from a diseased chamois found in 2001 in the Alt Pallars-Aran NHR. Sera from CAUBS were also tested with BDV-4 strain Cadí-6 [14]; (Gen Bank accession number AM905923), isolated in the Cadí NHR in 2006. The biotype of all virus strains used was non-cytopathogenic.

The VNT were performed according to the procedure described in the Manual of Diagnostic Tests and Vaccines for Terrestrial Animals [15] using Madin–Darby bovine kidney (MDBK) cells. Neutralizing antibody titres were expressed as the reciprocal of the highest dilution that neutralized 100 tissue culture infective doses (100 TCID_50_) in all cultures, calculated using Reed and Muench’s method [16]. Titres of 1∶10 and higher were considered positive. Viral replication was monitored by the Immuno-Peroxidase Monolayer Assay (IPMA) with polyclonal home-made pestivirus-specific serum.

### Virus Detection

Reverse transcription-polymerase chain reaction (RT-PCR) in wild ruminant samples was performed in spleen homogenates or in sera when no spleen samples were available. RT-PCR was performed using described panpestivirus primers 324 and 326 [17] and a commercial kit (One-Step PCR kit, Qiagen, Hilden, Germany). Before the RT-PCR, viral RNA was extracted using a commercial kit (Nucleospin Viral RNA Isolation, Macherey Nagel, Düren, Germany). Domestic ruminant sera were tested for the presence of pestiviral antigen with a commercial sandwich ELISA assay (Synbiotics, Lyon, France) according to the manufacturer’s procedure. This assay detects the NS3 protein and allows identification of immunotolerant persistently infected animals, but it can also detect transient infected animals. The validation report provided by the manufacturer reports a sensitivity and specificity index ‘approaching 100%’ in sera samples from cattle [18].

### Sequence Analysis

Sequence analyses of the 243 bp fragment of the 5′UTR region from RT-PCR positive samples were performed using primers 324 and 326 [17]. Purified amplicons (Minelute Gel Extraction Kit, Qiagen, Hilden, Germany) were analyzed with Big Dye Terminator v.3.1 Kit and the ABI 3130xl Genetic Analyzer (Applied Biosystems, Warrington, United Kingdom). The phylogenetic tree was constructed by the neighbour-joining method [19] using automatic root location. A bootstrap analysis of 1,000 replicates was performed by creating series of bootstrap samples to test tree branch reliability.

### Statistical Analysis

We calculated true prevalence and the confidence interval for single proportion calculations (exact binomial method) [20]–[21] in all the studied species in each zone. We stratified the prevalence in chamois in terms of the year. We classified the chamois from each study area into two groups according to whether they were born before/during the outbreak (2001–2002 for VAPS and 2005–2006 for CAUBS) or afterwards. We calculated the prevalence in each of these groups using the methods described above.

In order to test for differences in the observed proportions of positive Pyrenean chamois in VAPS and CAUBS we computed a chi-squared and reported the odds ratio (OR) values with its 95% confidence interval. The same test was computed to test for differences in chamois in terms of their date of birth with respect to the pestivirus outbreak (before/during or after) and to test for differences according to sex. We also used this test to check for differences between the two study areas in the observed proportions of positive animals in the rest of the studied species. A P-value for the chi-squared statistic <0.05 was interpreted as a lack of homogeneity between the proportions and therefore as a statistically significant difference between the two groups.

We performed a Wilcoxon signed-rank test to compare the affinity of the antibodies against the tested viral strains. To test for differences in the mean ranks we compared pairwisely the antibody titres against each virus strain. The limit of statistical significance was defined as P<0.05.

Due to the small number of samples tested by VNT, in wild ruminants other than Pyrenean chamois we checked each animal’s titre individually in order to detect greater than two-fold differences against different strains. The OIE has defined a ‘rule’ whereby a three-fold difference or more between end-points of two titrations should be regarded as decisive for infection by the virus species yielding the highest titre [15].

For data analysis we used the functions for analysing epidemiological data included in the library ‘epiR’ (http://cran.r-project.org/web/packages/epiR/epiR.pdf/) and the free statistical software R (http://www.r-project.org/). In this R package point estimates and confidence intervals are based on formulae provided by Rothman [20].

## Results

### Serological Results

Specific pestivirus antibodies were detected in Pyrenean chamois (prevalence in VAPS was 73.47% (95% CI 63.93–81.21) and in CAUBS 52.79% (95% CI: 43.30–62.06)), red deer (prevalence in VAPS was 10.73% (95% CI: 4.11–23.71) and in CAUBS 15.15% (95% CI: 5.45–33.99)) and roe deer (prevalence in VAPS was 1.13% (95% CI: 0.00–10.21) and in CAUBS 3.58% (95% CI: 0.00–21.00)) from both study areas, as well as in fallow deer (prevalence was 8.60% (95% CI: 1.66–28.19)) and European mouflon (prevalence was 19.19% (95% CI: 0.02–62.06)) from VAPS. Antibodies were also detected in sheep (prevalence in VAPS was 23.12% (95% CI: 18.96–27.83) and in CAUBS 49.49% (95% CI: 42.27–56.71)), goat (prevalence in VAPS was 13.47% (95% CI: 9.68–18.32) and in CAUBS 32.65% (95% CI: 23.94–42.67)) and cattle (prevalence in VAPS was 70.34% (95% CI: 65.39–74.84) and in CAUBS 65.94% (95% CI: 58.58–72.59)) from both zones. Prevalence values from all these species, along with prevalence in Pyrenean chamois per year, are given in Table 2. In VAPS prevalence in chamois remained constant during the study period, while in CAUBS it tended to fall. Prevalence was also calculated in chamois born before/during the outbreak and in chamois born afterwards. From these two groups, however, only 35 chamois from VAPS and 84 chamois from CAUBS could be classified, since age data were not always available. The prevalence in animals born before or during the outbreak was 77.04% (95% CI: 56.12–89.77) in VAPS and 64.43% (95% CI: 52.70–74.62) in CAUBS, while the prevalence in chamois born after the epizooty was 61.14% (95% CI: 34.87–82.11) in VAPS and 14.52% (95% CI: 3.35–41.65) in CAUBS.

**Table 2:** Prevalence of pestivirus antibodies in the analyzed species.

		Prevalence [95% CI]		Prevalence [95% CI]	
	Year of sampling	VAPS	n	CAUBS	n
Pyrenean chamois	2007	62.12% [29.87–86.17]	8	19.19% [0.02–62.06]	5
	2008	53.37% [28.42–76.55]	13	69.25% [48.62–84.23]	23
	2009	52.46% [30.26–73.57]	17	68.91% [49.50–83.33]	26
	2010	74.74% [52.65–88.70]	20	44.17% [29.44–59.89]	38
	2011	90.14% [77.22–96.10]	41	32.65% [14.31–57.86]	15
	Total	73.47% [63.93–81.21]	99	52.79% [43.30–62.06]	107
Red deer	Total	10.73% [4.11–23.71]	43	15.15% [5.45–33.99]	25
Roe deer	Total	1.13% [0.00–10.21]	47	3.58% [0.00–21.00]	22
Fallow deer	Total	8.60% [1.66–28.19]	21	ND	0
Mouflon	Total	19.19% [0.02–62.06]	5	ND	0
Sheep	Total	23.12% [18.96–27.83]	360	49.49% [42.27–56.71]	184
Goats	Total	13.47% [9.68–18.32]	258	32.65% [23.94–42.67]	96
Cattle	Total	70.34% [65.39–74.84]	361	65.94% [58.58–72.59]	175

Prevalence and 95% confidence interval (exact 95% binomial confidence intervals) of the pestivirus NS3 antibodies in all the analyzed species. In the Pyrenean chamois prevalence is classified according to hunting season.

CI = Confidence interval.

n = number of tested animals.

ND = not done.

VAPS = Val d’Aran and Pallars Sobirà regions.

CAUBS = Cerdanya, Alt Urgell and Berguedà regions.

VNT was performed on 81 chamois, 10 red, one roe and two fallow deer, one mouflon, 133 sheep, 67 goats and 84 cattle sera. Mean titres for Pyrenean chamois, sheep, goats and cattle, along with their median and range values, appear in Table 3. VNT results in goats from VAPS showed the greatest individual variability in comparison with cattle and sheep from both study areas and goats from CAUBS. For that reason we reported the VNT results from the goats from VAPS individually (Table 4). Titres were compared following the OIE’s ‘rule’, searching for more than two-fold differences between end-points different titrations, [15]. Several goats had higher antibody titres to the BVDV strain (n° 1–11), others to the BDV strains (n° 12–17) and the rest showed no clear differences between the titres against BVDV and BDV. VNT results from wild ruminants other than Pyrenean chamois are given individually in Table 5. Several red deer (n° 1, 2, 8, 9) had BDV specific antibodies but no conclusive differences were detected between different BDV strains. Red deer n°10 had specific antibodies against both BDV-4 strains and red deer n° 4 against the BDV-Aran. Red deer n° 6 and 7 had higher titres against the BVDV strain and in the rest of red deer no differences were detected in the titres between BDV and BVDV. The VNT performed on roe deer n° 1 showed no conclusive differences between the titres against BVDV and BDV. Fallow deer n° 1 and 2 had had BDV-specific antibodies, but no determinant differences were found between different BDV strains.

**Table 3:** Antibody titres from the Pyrenean chamois, sheep, goats and cattle against five pestivirus strains.

		BVDV-NADL			BDV-Esp97			BDV-Aran-1/Cadí-6^a^			BDV-137/4			
STUDY AREA	SPECIES	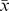	Md	range	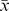	Md	range	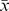	Md	range	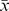	Md	range	n
	Chamois	73.12	40	0–640	254.06	160	10–1280	581.87	160	20–10240	784.37	320	20–5120	32
**VAPS**	Sheep	60.88	20	0–640	242.53	80	0–2560	191.77	80	0–1280	234.12	20	0–5120	79
	Goat	113.71	20	0–640	138	20	0–2560	104.85	10	0–1280	21.42	0	0–160	35
	Cattle	664.11	320	10–5120	161.37	40	0–2560	71.56	20	0–1280	107.64	10	0–2560	51
	Chamois	165.51	20	0–5120	297.95	160	0–2560	447.34	320	40–1280	410.61	80	20–10240	49
**CAUBS**	Sheep	70.37	40	0–640	173.88	160	10–640	167.22	160	10–640	121.29	80	0–1280	54
	Goat	177.5	80	20–1280	160.62	120	20–640	203.75	160	40–640	105.62	60	10–640	32
	Cattle	542.12	160	10–5120	128.48	40	0–1280	37.27	20	0–160	16.96	10	0–80	33

Mean, median and range (min-max) of the antibody titres from Pyrenean chamois, sheep, goat and cattle against one *Bovine viral diarrhoea virus* (BVDV) strain of type 1 (BVDV-NADL), three *Border disease virus* (BDV) strains of type 4 (BDV-Esp97, -Aran-1 and -Cadí-6) and one BDV strain of type 1 (BDV-137/4).

a BDV-Aran-1 was tested against sera from VAPS, while BDV-Cadí-6 was tested against sera from CAUBS.

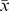
 = Arithmetic mean.

Md = Median.

CI = Confidence interval.

n = number of tested animals.

VAPS = Val d’Aran and Pallars Sobirà regions.

CAUBS = Cerdanya, Alt Urgell and Berguedà regions.

**Table 4:** Individual antibody titres from the analyzed goats from VAPS against four pestivirus strains.

Goat	BVDV-NADL	BDV-Esp97	BDV-Aran-1	BDV-137/4
1	640	80	40	10
2	80	10	10	10
3	20	0	0	0
4	20	0	0	0
5	80	0	0	0
6	20	0	0	0
7	20	0	0	0
8	20	0	0	0
9	20	0	0	0
10	320	20	10	20
11	20	0	0	0
12	0	20	320	10
13	80	80	1280	40
14	0	20	160	20
15	40	2560	20	20
16	0	160	80	80
17	0	40	10	0
18	640	160	10	10
19	640	640	320	160
20	10	10	0	0
21	640	320	320	160
22	20	10	0	20
23	80	160	640	40
24	20	0	20	0
25	80	320	320	80
26	160	40	10	0
27	40	10	0	0
28	80	20	10	0
29	10	10	0	0
30	20	20	0	0
31	10	20	10	0
32	80	20	0	0
33	40	0	0	10
34	20	40	40	20
35	10	40	40	40

Antibody titres of 35 goats from VAPS against one *Bovine viral diarrhoea virus* (BVDV) strain of type 1 (BVDV-NADL), two *Border disease virus* (BDV) strains of type 4 (BDV-Esp97 and -Aran-1) and one BDV strain of type 1 (BDV-137/4). Neutralizing antibody titres are expressed as the reciprocal of the highest dilution that neutralized 100 tissue culture infective doses (100 TCID_50_) in all cultures.

**Table 5:** Individual antibody titres from the analyzed red, fallow and roe deer and mouflon against five pestivirus strains.

	Study area	Samplingdate	BVDV-NADL	BDV-Esp97	BDV-Aran/Cadí-6^a^	BDV-137/4
Red deer n° 1	VAPS	2007	0	80	20	40
Red deer n° 2	VAPS	2008	10	20	80	20
Red deer n° 3	VAPS	2009	320	20	320	0
Red deer n° 4	VAPS	2009	10	0	320	0
Red deer n° 5	VAPS	2010	1280	320	80	2560
Red deer n° 6	CAUBS	2007	10	0	0	0
Red deer n° 7	CAUBS	2008	10	0	0	0
Red deer n° 8	CAUBS	2009	20	80	160	20
Red deer n° 9	CAUBS	2009	0	160	160	160
Red deer n° 10	CAUBS	2010	0	20	80	0
Roe deer n° 1	CAUBS	2009	160	20	20	80
Fallow deer n° 1	VAPS	2008	10	80	40	80
Fallow deer n° 2	VAPS	2008	10	160	40	80
Mouflon n° 1	VAPS	2010	640	160	160	2560

Individual antibody titres of positive red, fallow and roe deer and mouflon against one *Bovine viral diarrhoea virus* (BVDV) strain of type 1 (BVDV-NADL), three *Border disease virus* (BDV) strains of type 4 (BDV-Esp97, -Aran-1 and -Cadí-6) and one BDV strain of type 1 (BDV-137/4). Neutralizing antibody titres are expressed as the reciprocal of the highest dilution that neutralized 100 tissue culture infective doses (100 TCID_50_) in all cultures.

a Sera from VAPS were tested against BDV-4 strain Aran, while sera from CAUBS were tested against BDV-4 strain Cadí-6.

VAPS = Val d’Aran and Pallars Sobirà regions.

CAUBS = Cerdanya, Alt Urgell and Berguedà regions.

### Prevalence Related Factors

Seroprevalence was significantly higher in chamois from VAPS (OR_VAPS_ = 2.46; 95% CI: 1.36–4.42) than in chamois from CAUBS. In CAUBS, the risk of being seropositive was significantly higher in chamois born before or during the outbreak (OR_Born before/during_ = 10.12; 95% CI: 2.07–49.30) than in chamois born after the outbreak. However, no significant differences were observed between these groups in VAPS. No significant differences were detected in the prevalence between chamois sexes in either study area.

No differences were detected between the study areas in prevalence in either red or roe deer. In sheep and goats seroprevalence was significantly higher in CAUBS (OR_sheep CAUBS_ = 3.18; 95% CI: 2.18–4.64; OR_goats CAUBS_ = 2.98; 95% CI: 1.72–5.17) than in VAPS. No differences were detected between the study areas in the prevalence in cattle.

### Comparative VNT

Six pairwise combinations between viruses used in VNT were tested in each ruminant species analyzed. Table 6 shows the significant differences (P<0.05) between pairwise comparisons. Pyrenean chamois from VAPS had significantly higher titres against the BDV strains than against the NADL strain; however, no significant differences were detected between the mean titres against the different BDV strains. The chamois from CAUBS had significantly higher titres against the strain of chamois origin, BDV-Cadí-6. In VAPS, the sheep had significantly higher titres against both BDV-Esp97 and BDV-Aran-1, the goats against both BVDV-NADL and BDV-Esp97, and cattle against BVDV-NADL. One sheep showed null titres against all the strains and was considered as an ELISA false positive. In CAUBS, the comparison of means ranks determined that sheep had significantly higher antibody titres against both BDV-Esp97 and BDV-Cadí-6, goats against BVDV-NADL, BDV-Esp97 and BDV-Cadí-6, and cattle against BVDV-NADL.

**Table 6:** Significant differences between titres against different strains in sheep, goat, cattle and Pyrenean chamois in the two study areas.

	VAPS	CAUBS
BVDV-NADL vs. BDV-137/4	G, Ca, Ch	S, G, Ca, Ch
BVDV-NADL vs. BDV-Esp97	S, Ca, Ch	S, Ca, Ch
BVDV-NADL vs. BDV-Aran-1/Cadí-6^a^	S, G, Ca, Ch	S, G, Ca, Ch
BDV-137/4 vs. BDV-Esp97	S, G	S, G, Ca
BDV-137/4 vs. BDV-Aran-1/Cadí-6	S, G	S, G, Ca, Ch
BDV-Esp97 vs. BDV-Aran-1/Cadí-6	–	Ch

Significant differences between pairwise comparisons in titres against five pestivirus strains (one *Bovine viral diarrhoea virus* (BVDV) strain of type 1 (BVDV-NADL), three *Border disease virus* (BDV) strains of type 4 (BDV-Esp97, -Aran-1 and -Cadí-6) and one BDV strain of type 1 (BDV-137/4)) in sheep, goat, cattle and Pyrenean chamois in the two study areas.

S = Sheep.

G = Goat.

Ca = Cattle.

Ch = Chamois.

a Sera from VAPS were tested against BDV-4-Aran-1, while sera from CAUBS were tested against BDV-4-Cadí-6.

### Virological Results

**Figure 2:**
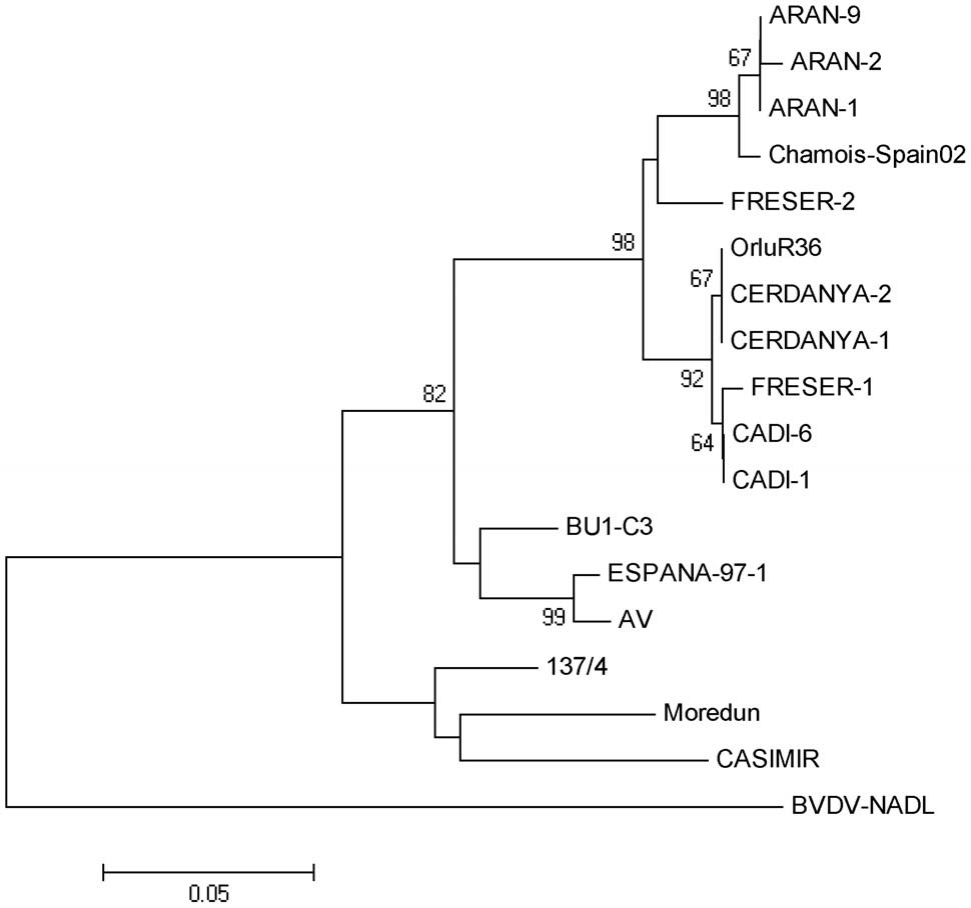
Unrooted neighbour-joining phylogenetic tree based on the 5′UTR sequence among pestiviruses. The strains detected in six chamois (ARAN-9, -10, -11.-12, -13, and -14) showed genetic identity and subsequently the analysis was performed only with one sequence (ARAN-9). The detected strains cluster with other chamois viruses isolated in the bordering NHR of Alt Pallars-Aran (ARAN-1, and -2). The numbers on the branches indicate the bootstrap values (in percentage; 1,000 replicates). Sequences of strains were taken from Gen Bank with following accession numbers: ARAN-9 (HE818617), ARAN-10 (HE818618), Aran-11 (HE818619), ARAN-12 (HE818620), ARAN-13 (HE818621), ARAN-14 (HE818622), ARAN-1 (AM765800), ARAN-2 (AM765801), Chamois-Spain02 (AY641529), FRESER 2 (FN691777), ORLUR36 (DQ898294), CERDANYA-2 (AM905931), CERDANYA-1 (AM905930), CADI-6 (AM905923), CADI-1 (AM905918), BU1-C3 (DQ361068), Espana-97 -1 (FR714860), AV (EF693984), 137/4 (U65052), Moredun (U65023), CASIMIR (AB122085), BVDV NADL (M31182). The sequence of strain FRESER-1 is not deposited in the GenBank. The scale bar represents 0.05 substitutions per nucleotide site.

Viral RNA was detected by RT-PCR in six (viroprevalence 5.26%; 95% CI: 2.43–11.00) apparently healthy hunted chamois from VAPS: a five-year-old male, an adult male, an old male and two females (exact age unreported); the sex and age of the sixth animal was not reported. Sequences from viruses found in these animals were named ARAN-9, -10, -11, -12, -13 and -14 and were deposited in the Gen Bank under following accession numbers: HE818617, HE818618, HE818619, HE818620, HE818621, and HE818622. The strains ARAN-9, -10, -11.-12, -13, and -14 showed genetic identity and subsequently the phylogenetic analysis was performed only with one sequence (ARAN-9). This late strain appears in the phylogenetic tree of figure 2 and it clusters with other chamois viruses isolated in the bordering NHR of Alt Pallars-Aran (ARAN-1, and -2) in 2001. No viruses were detected in chamois from CAUBS or in any of the other species in either study areas.

## Discussion

This study focuses on the first two areas severely affected by outbreaks of BDV infection that were associated with high mortality in Pyrenean chamois. Unexpectedly, we found different scenarios in each population.

The prevalence of pestivirus antibodies in Pyrenean chamois was high in both study areas. However, prevalence was higher in VAPS than in CAUBS and remained stable during the post-outbreak years; by contrast, in CAUBS prevalence tended to decrease after the outbreak. The remarkable prevalence of antibodies in 2011 in VAPS (90.14%) is the highest prevalence ever described in a Pyrenean chamois population. These results suggested that high circulation of pestiviruses in Pyrenean chamois occurs in VAPS, which concurs with the results from the years immediately after the outbreak (2002–2004) [10]. Interestingly, when analysing the effect of year of birth, in VAPS we found the same prevalence in chamois born before and after the outbreak, which reinforces the idea that high circulation of BDV has existed in this area since the disease outbreaks in 2001 and 2002. However, in CAUBS, the lower prevalence in the chamois born after the outbreak suggests that the circulation of pestiviruses in this area has fallen since the high mortality recorded between 2005 and 2006.

The VNT results for chamois showed that these animals possessed specific BDV antibodies in both study areas. However, in VAPS no significant differences were found between the different border strains, while in CAUBS the chamois were found to have significantly higher titres against BDV-Cadí-6, the strain originating from the chamois. This result suggests that these animals were infected during the outbreak but then seroconverted and cleared themselves of the virus. It is interesting to note that all the chamois found to be clinically affected by the disease were antibody negative and died without seroconverting [10].

In VAPS, the absence of significant differences between the different BDV strains could be due to serological cross-reactivity, which has been reported as occurring between all members of the genus Pestivirus [22]. Nevertheless, it is more likely that this lack of significant differences is related to the greater length of time elapsing since the outbreak in VAPS than in CAUBS (2001 and 2002 vs. 2005 and 2006). Even though antibodies against pestivirus are said to remain detectable for as long as an animal survives, it seems that with time it becomes more difficult to determine the pestivirus species that cause infection. In addition, as described above, the OIE has defined a ‘rule’ whereby a three-fold difference or more between end-points of two titrations should be regarded as decisive for infection by the virus species yielding the highest titre [15]. However, to the authors’ knowledge, no ‘rule’ exists when comparing different subgroups of the same pestivirus species or even the same genotype (e.g. the BDV-4-Esp97 and BDV-4 strain chamois).

Antibodies against pestivirus were detected in all the studied wild ruminants: red, roe and fallow deer and mouflon. Interestingly, Marco *et al.* [23] did not detect antibodies against pestivirus in these species during a study performed after the disease outbreak in the Alt Pallars-Aran NHR (part of the VAPS study area), most probably due to their small sample size.

In red deer the apparent prevalence was low (10.73% in VAPS and 15.15% in CAUBS). Several studies have detected antibodies against pestivirus in other areas, generally associated with BVDV infection [24]–[25], and a previous study detected a low seroprevalence in red deer from the Pyrenees [23]. Unexpectedly, VNT results showed that the majority (6/10) of the red deer (n° 1, 2, 4, 8, 9, 10) had specific antibodies against BDV strains (the end-points of titrations against a BDV strain were three-fold or higher when compared to the BVDV strain). Red deer n° 10 had specific antibodies against both BDV-4 strains, suggesting that it had been infected with this BDV genotype. The finding of a red deer (n° 4) from CAUBS with a greater than two-fold higher titre against BDV-4-Cadí-6 is remarkable. This animal was probably infected during the disease outbreak that occurred in chamois between 2005 and 2006. Red deer n° 6 and 7 had specific BVDV antibodies, while n° 3 and 5 showed no differences in the titres between BDV and BVDV. This could be due to cross-reactivity or the result of a past pestivirus infection and subsequent contact with a different pestivirus species.

The prevalence in roe deer from both study areas was very low (1.13% in VAPS and 3.58% in CAUBS). Although higher prevalences have previously been reported, studies in the Pyrenees and the Alps have failed to detect antibodies in this species [23]–[28]. The VNT performed on one roe deer showed no conclusive differences between the titres against BVDV and BDV and so it is not possible to determine which pestivirus species infected this animal.

Prevalence in fallow deer in VAPS was also low (8.60%). Other studies have also failed to detect pestivirus antibodies in fallow deer [25], [29]. The VNT of two positive animals showed that they had BDV-specific antibodies, but no determinant differences were found between different BDV strains.

Deer species are more likely to have antibodies against BVDV [5] and indeed only BVDV has been isolated in red [6], roe [30]–[33] and fallow deer [34]. The predominance of BDV-specific antibodies in cervids in our study is likely to be related to the high circulation of BDV in chamois populations during the outbreaks, which would have facilitated a spill-over of BDV from chamois to other species.

We detected an antibody-positive mouflon, but the sample size was too small to assess the infection status in this species. Marco *et al*. [23] also found low seroprevalence in mouflons from the same area, although in other areas such as the Alps high seroprevalence has been reported [35].

The low prevalence of antibodies in wild ruminants (other than Pyrenean chamois) suggests that they do not play a key role in maintaining BDV in these populations. However, the high circulation of BDV in Pyrenean chamois during the disease outbreaks seems to lead to an occasional spill-over of BDV to other wild ruminants, which then play the role of ‘victims’.

The seroprevalence found in sheep from VAPS (23.12%) was lower than that previously reported from this area (64–69%) [23], [36]. However, seroprevalence in CAUBS (49.49%) was significantly higher than in VAPS. BD is widespread in sheep from Spain, even though clinical disease and virus isolation have only been reported in recent years [37]–[38]. Sheep from VAPS and CAUBS had similar titres against both BDV-Esp 97 and BDV-4 originating from chamois. There are two possible explanations for these results. The first is that the sheep were infected by both strains, since both strains circulate in Spain. The second is that sheep were infected with one of both strains long time ago and, the lapse of time since the infection has increased cross-reactivity of antibodies between the two BDV-4 strains [15].

Prevalence in goats was significantly higher in CAUBS than in VAPS (32.65% vs. 13.47%) and both values are higher than previously described in the Pyrenees (7.3%) [23]. In this species, no significant differences between the mean ranks of the titres against BDV-Esp97 and BVDV-NADL were detected. This is due to the fact that some goats showed of our study showed specific BDV antibodies while others had specific BVDV antibodies. As shown in table 4, we noticed that several goats have higher antibody titres to the BVDV strain (n° 1–11), others to the BDV strains (n° 12–17) and the rest showed no clear differences between the titres against BVDV and BDV. It has been suggested that sheep and cattle are the main origin of pestivirus infection in goats [39]–[40]. Interestingly, we found three goats with specific antibodies against BDV-Aran (n° 12, 13 and 14), which suggests that not only sheep and cattle but also Pyrenean chamois can act as a source of infection for goats.

Although several studies have suggested that sheep could be a source of BDV infection for chamois [23], [35], our results do not allow us confirm or refute this idea. Nevertheless, the seroprevalence in sheep and goats in both areas is moderate, suggesting that pestiviruses circulate in these species. Given that these animals cohabit with chamois, it is possible that pestivirus exchanges occur. However, if we are to assess with greater accuracy the role of domestic ruminants in the epidemiology of BDV infection in chamois, more studies are needed to isolate the BDV strains circulating in sheep and goats in the Pyrenees.

The prevalence detected in cattle is high in both areas and specific titres are present against BVDV. However, vaccination in this species is a routine event, which could explain the high seroprevalence, as has previously been described [23]. Despite the fact that vaccinating is commonplace (and usually unreported by veterinarians), our results suggest that BVDV circulates frequently, since in VNT we detected 11/35 individual goats with more than two-fold titres against BVDV-1-NADL.

Viral RNA was detected in six Pyrenean chamois, all from VAPS. Viral sequencing determined that they were infected with a BDV-4. This fact reinforces our previous hypothesis that BDV have circulated in VAPS ever since the severe outbreak of disease but may not circulate – or only at a low level – in CAUBS. These differences, together with the other differences mentioned above, reveal the existence of two different epidemiological scenarios for BDV infection in the aftermath of the severe outbreaks of disease that decimated chamois populations.

It is difficult to determine the cause of the differences between the two studied areas. During the BDV epizooty, the highest mortality values in the whole of the Pyrenees were recorded in CAUBS (85.6%) [4]. This extremely high mortality rate could have endangered the long-term maintenance of the BDV in the chamois population given the reduction in the number of animals susceptible to infection. Census data reveal that the recovery in this population after the outbreak was quick. However, our results suggest that there are an increasing proportion of naïve chamois that could provoke a second BDV outbreak in the future.

In VAPS, the chamois mortality described during the BDV outbreak was lower than in CAUBS (42%). However, in VAPS chamois numbers have not recovered as they have in CAUBS. This coincides with the isolation of BDV in chamois from this area and suggests that BDV infection does have a negative impact on chamois population dynamics. More studies are still needed if we are to assess the role of BDV in the decline of this chamois population, which could be associated with low survival and fecundity rates as occurs in Pestivirus infection in domestic ruminants [6].

It is worth remarking that the chamois population from VAPS could be a source of BDV in other chamois populations. Indeed, a recent study has linked a BDV outbreak between 2009 and 2010 in Pyrenean chamois in Andorra with the arrival of BDV viruses from the VAPS population [41]. In addition, in 2011 the first clinical cases of BDV infection were described from the Spanish region of Aragon, which borders on VAPS to the west.

The lack of viral detection in domestic and wild ruminants other than Pyrenean chamois was expected. In domestic ruminants, the short viraemia in acute infections and the low prevalence of PI animals makes it very difficult to detect infected animals [42], while in wild ruminants the low prevalence observed suggests very low virus circulation that hampers greatly the detection of viraemic animals.

Our study confirms that the BDV infection outbreaks in the Pyrenees only affected the Pyrenean chamois, although other wild ruminant species can occasionally be infected. After the severe BDV outbreaks, at least two different scenarios appeared in the Pyrenees: on the one hand, in some areas the disease has become endemic and BDV circulates frequently in the chamois population, possibly having a negative impact on host population dynamics, while, on the other hand, in other areas BDV does not seem to circulate and to affect host population dynamics. Thus, management of chamois populations affected by these epizootics should be designed according to the epidemiological status and no generalizations should be made.
